# The utilization of artificial intelligence in glaucoma: diagnosis versus screening

**DOI:** 10.3389/fopht.2024.1368081

**Published:** 2024-03-06

**Authors:** Mo’ath AlShawabkeh, Saif Aldeen AlRyalat, Muawyah Al Bdour, Ayat Alni’mat, Mousa Al-Akhras

**Affiliations:** ^1^Department of Ophthalmology, Al Taif Eye Center, Amman, Jordan; ^2^Department of Ophthalmology, The University of Jordan, Amman, Jordan; ^3^Department of Ophthalmology, Houston Methodist Hospital, Houston, TX, United States; ^4^Department of Computer Information Systems, School of Information Technology and Systems, The University of Jordan, Amman, Jordan

**Keywords:** glaucoma, artificial intelligence, screening, diagnosis, deep learning

## Abstract

With advancements in the implementation of artificial intelligence (AI) in different ophthalmology disciplines, it continues to have a significant impact on glaucoma diagnosis and screening. This article explores the distinct roles of AI in specialized ophthalmology clinics and general practice, highlighting the critical balance between sensitivity and specificity in diagnostic and screening models. Screening models prioritize sensitivity to detect potential glaucoma cases efficiently, while diagnostic models emphasize specificity to confirm disease with high accuracy. AI applications, primarily using machine learning (ML) and deep learning (DL), have been successful in detecting glaucomatous optic neuropathy from colored fundus photographs and other retinal imaging modalities. Diagnostic models integrate data extracted from various forms of modalities (including tests that assess structural optic nerve damage as well as those evaluating functional damage) to provide a more nuanced, accurate and thorough approach to diagnosing glaucoma. As AI continues to evolve, the collaboration between technology and clinical expertise should focus more on improving specificity of glaucoma diagnostic models to assess ophthalmologists to revolutionize glaucoma diagnosis and improve patients care.

## Overview

Artificial intelligence has ushered in a transformative era in ophthalmology and continues to be implemented in different disciplines in ophthalmology practice, especially in the domain of glaucoma (1). Diagnosis models, tailored with precision and specificity, are proving indispensable in specialized ophthalmology clinics. These models are designed to delve deep into complex datasets, accounting for a myriad of patient-specific variables to ensure accurate and timely diagnosis. In contrast, screening models, streamlined for efficiency and broader applicability, are being employed in general practice settings. Their primary objective is to flag potential cases among a larger pool, serving as an initial sieve or triage for community referrals to capture those who require a more detailed assessment and to classify who should see a glaucoma specialist and who should not in order to prioritize care and hence improve healthcare outcomes (2). Both types have generally gained significant attention due to their potential to enhance the diagnostic process by identifying subtle changes in the optic nerve and retinal structures that might not be discernible to human eyes and also by providing rapid and consistent assessments of optic nerve damage. Moreover, they have the potential to improve accessibility to diagnosis in underserved regions where expert ophthalmologists are scarce (3). The bifurcation of these AI applications – diagnosis for specialty clinics and screening for general practice – accentuates the diverse utility of machine learning and deep learning in catering to both depth and breadth in glaucoma care. In this review, we will provide a review on how diagnostic model development is different from screening model development in glaucoma.

## Diagnostic and screening models: sensitivity vs. specificity

**Figure 1:**
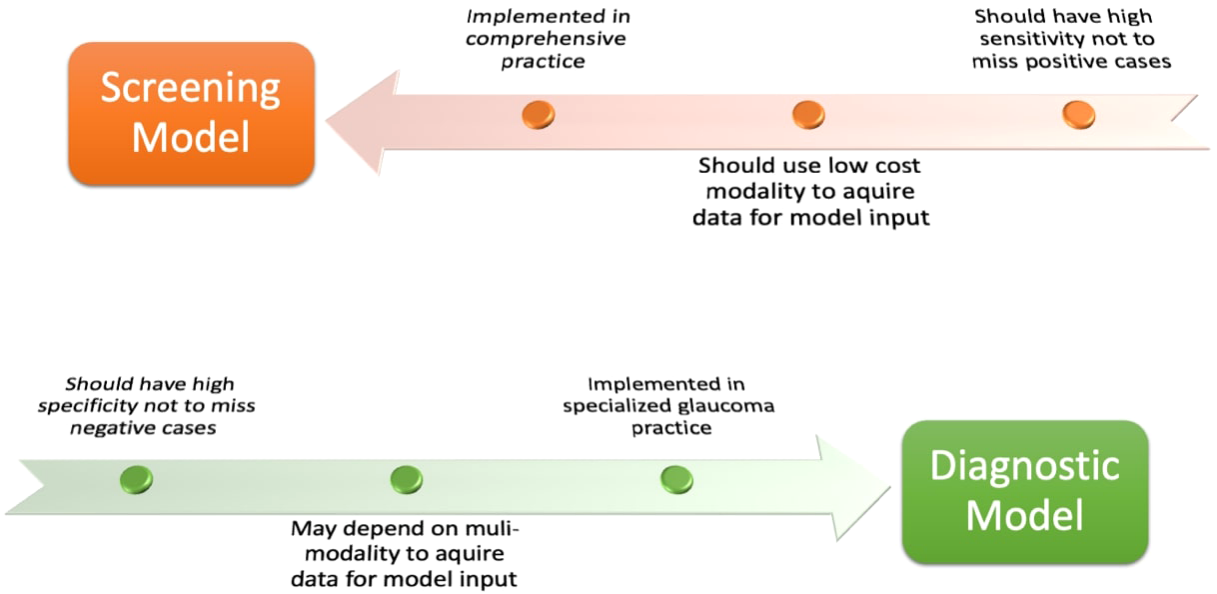
Screening model versus diagnostic model.

In medical diagnostics, the balance between sensitivity and specificity is pivotal, and AI models developed for glaucoma are no exception. For screening models deployed in general practice settings, sensitivity takes precedence. A highly sensitive model ensures that as many potential cases of glaucoma as possible are identified, even at the risk of including some false positives, because as the economic and personal impacts of glaucoma tend to worsen with further progression to advanced disease, early detection and accurate diagnosis followed by prompt treatment are of vital importance to prevent vision loss and the subsequent burden associated with glaucoma (4). Conversely, in specialized ophthalmology clinics, diagnostic models should prioritize specificity. A specific diagnostic tool aims to confirm the disease with a high degree of accuracy, reducing the likelihood of false positives (**Figure 1**). By minimizing erroneous diagnoses, resources can be efficiently utilized, unnecessary treatments can be avoided, and patients can be reassured of the accuracy of their diagnosis. This diagnostic process relies on judgment on the combination of abnormalities detected upon precise evaluation of many aspects, including optic nerve appearance, particularly optic disc cupping, intraocular pressure measurement, visual field (VF) assessment as well as assessment of structural optic nerve injury using various imaging modalities, most importantly optical coherence tomography (OCT) imaging (5). In addition, documentation of progression in these parameters through patients’ follow-up is an essential factor in the diagnosis. Together, the emphasis on sensitivity in screening and specificity in diagnosis ensures a holistic and tiered approach to glaucoma management through AI.

## Screening models

Screening models for glaucoma have been tailored predominantly around one of the hallmark clinical features of the condition: the cup-to-disc ratio from colored fundus photographs. Other screening models utilize retinal nerve fiber layer thickness for OCT imaging or visual field tests as well. Colored fundus photographs are considered the simplest and the most widely adopted method for visualization and examination of the optic nerve head (6). They provide an easy, non-invasive and relatively cost-effective tool not only for glaucoma screening but also for early detection, disease monitoring and patient education. However, the associated need for human expertise to interpret and grade the images, inter-observer discrepancies and anatomical variations of the optic nerve head appearance may negatively affect the diagnostic accuracy (2). For the sake of simplicity and clarity in differentiation, images with a large cup-to-disc ratio, indicative of potential glaucomatous changes, are often set in contrast to those with a small ratio, representing the controls (7). This clear demarcation aids the AI in identifying and flagging suspicious cases efficiently. Additionally, given the crucial nature of early detection in glaucoma, it is imperative that these screening models yield high sensitivity. By doing so, they prioritize capturing as many potential glaucoma cases as possible, even if it means occasionally mislabeling a healthy eye. The overarching philosophy behind this approach is that it is preferable to over-identify and then refine through further assessment rather than miss a case and risk irreversible vision loss for a patient.

In this review methodology, we conducted a comprehensive literature review by performing a PubMed search using keywords (Glaucoma, Artificial Intelligence, Screening, Diagnosis, Deep Learning), and we have established rigorous eligibility criteria for article selection, emphasizing relevance to our research topic, recent publication dates, peer-reviewed status, credibility of the source, and scholarly authority. This ensured the inclusion of high-quality and pertinent literature in our review. Next, the AI models were categorized into screening models and diagnostic ones according to the above-mentioned indicators.

## Models using colored fundus photographs

Observing changes in the optic nerve and retinal structures and monitoring the cup-to-disc ratio over a period of time can automate the screening process. The observation using automated image processing and AI tools can aid in screening and potentially patient self-testing to check their retinas regularly. If a change in the cup-to-disc ratio is observed, the patient can be further referred for a more detailed diagnosis.

The majority of currently published studies are primarily screening studies. The first publication studying the application of AI on colored fundus photographs was back in 1999, when Sinthanayothin et al. described successful anatomical localization of the optic disc, fovea, and blood vessels from 112 retinal photographs with precise accuracy and high sensitivity (8). Later on, several authors have evaluated AI-based colored fundus photograph analysis for its utility for detecting glaucoma. Some of these publications developed AI models relying on segmentation and analysis of the optic nerve head (9–11) while others were based on detecting features suspicious for glaucomatous optic neuropathy such as high vertical cup-to-disc ratio, nerve fiber layer defects, peripapillary atrophy, disc hemorrhages and rim thinning (12–17) from colored fundus photograph datasets. All were proven to be efficient in screening for glaucomatous optic neuropathy with enough sensitivity to be transferred into real-world practice with an aim of improving patients care (18). Moreover, a similar algorithm was proposed to efficiently screen for closed-angle glaucoma, in which the authors trained their model to detect the hidden features of shallow anterior chamber on colored fundus photographs (19).

## AI models using OCT imaging of the optic nerve

In addition to colored fundus imaging, measurement of the retinal nerve fiber layer (RNFL) thickness is a critical component of glaucoma diagnosis; it offers an objective, quantitative, and early indicator of optic nerve damage, allowing for timely diagnosis, monitoring disease progression, and guiding treatment decisions to preserve a patient’s vision and quality of life (20) and therefore they were a major target for AI technologies (20). Several AI algorithms were developed and trained using OCT image datasets. These include Barella et al. (21) Burgansky-Eliash Z. et al. (22), Huang et al. (23), Naithani et al. (24), Xu J et al. (25), Larrosa et al. (26) and Muhammad et al. (27) which all have proved the effectiveness of AI systems in distinguishing healthy suspect eyes from eyes with early glaucoma and highlighted the potential role of these models in screening for glaucomatous abnormality with high sensitivity that can reach up to 100% according to some authors (28). Recently, Noury et al. proposed a DL model that was successfully trained to detect glaucoma in various ethnic groups based on OCT dataset analysis (29). Additionally, a new DL model was trained by anterior segment OCT dataset to function as an automated gonioscopy to screen for angle closure glaucoma at a valid accuracy (30).

## Models using visual field perimetry testing

When deep learning machines are trained by a large VF dataset and their models are processed to make VF features more susceptible to detection, AI algorithms can predict, diagnose and monitor glaucoma with high accuracy gains, low cost and raised efficiency (3). Since 1994, many publications have validated the efficiency of DL machines trained by standard automated perimetry (SAP) perimetry data in distinguishing glaucomatous VF patterns from normal and non-glaucomatous ones and in classifying the severity of glaucomatous field loss as in Goldbaum et al. (31). Chan et al. (32), Sample et al. (33), Andersson et al. (34), Bowd et al. (35), Cai et al. (36) and Kucur et al. (37). Furthermore, a smartphone application called “iGlaucoma” DL system was developed to interpret VF pattern deviation and was found to be a clinically effective tool to detect glaucomatous optic neuropathy showing its promise for clinical applicability (38).

## Diagnostic models

Diagnostic models for glaucoma delve deeper than their screening counterparts, focusing on a more comprehensive and definitive dataset. **Table 1** shows a comparison between the two models’ types. The gold standard for confirming glaucoma is the demonstration of longitudinal progression in glaucoma-pattern loss (39). Therefore, it is imperative that the data these models are trained on represents cases that have been unequivocally proven to exhibit this pattern over time. However, relying solely on structural changes might not suffice for a conclusive diagnosis. Incorporating multi-modality becomes pivotal in creating a robust diagnostic tool. A holistic evaluation of glaucoma ideally marries both functional and structural assessments. Functional imaging, like visual field tests, offers insights into the patient’s visual capabilities and potential areas of loss, providing a real-world context to the structural changes observed. Conversely, structural assessments, whether through colored fundus imaging or OCT, provide intricate details about the physical changes in the retina and optic nerve. By synergizing these modalities, diagnostic models offer a panoramic view of the disease’s state and progression, ensuring that the diagnosis is both accurate and holistic.

**Table 1:** Comparison between screening and diagnostic models in glaucoma.

Parameter	Glaucoma Screening Model	Glaucoma Diagnostic Model
**Use**	Initial identification of potential glaucoma cases.	Detailed assessment and confirmation of glaucoma.
**Data Used for Model Development**	Fundus images with varying cup to disc ratios.	Longitudinal data showcasing glaucoma-pattern loss.
**Nature of Data to be Inputted**	Predominantly fundus images with potential glaucomatous changes.	Multi-modal data, including visual field tests, fundus imaging, and OCT.
**Type of Model (IT Aspect)**	High-sensitivity detection models. Often simpler algorithms that prioritize breadth over depth. Simple image processing tools to observer cup to disc ratio changes over time can be utilized.	Advanced algorithms trained on multi-modal data. Typically utilizes deep learning and complex neural networks. Other classification models such as decision trees or support vector machines can be utilized in combination with image processing tools.
**Deployment**	General practice settings, primary care clinics, community health centers.	Specialized ophthalmology clinics, advanced diagnostic centers.

From an information technology (IT) perspective, a group of classifiers, normally referred to as an ensemble of classifiers, can be used to aid in the diagnosis process. Possible classifiers include, but are not limited to, neural networks, support vector machines and decision trees. It is either that each classifier might diagnose the case independently then their classifications are combined, either equally or weighted according to their accuracy, or an ensemble is constructed from multiple classifiers all receiving the same input. Such an approach does not only fine-tune the AI’s precision but it also fortifies the clinician’s confidence in the provided diagnosis. However, achieving complete independence among classifiers in an ensemble is difficult. Usually, the goal is to reduce the dependency as much as possible to ensure that the ensemble can benefit from the diversity of the classifiers. Some factors and conditions that could promote independence among classifiers and establish that the classifiers make their predictions without influencing each other in an ensemble are: using different data subsets, using different feature subsets, using fundamentally different algorithms, and random initialization of training parameters. Conducting statistical tests to check the correlation between the errors of the classifiers can provide empirical evidence for their independence.

While the previously mentioned models are primarily screening tools, a few were developed as diagnostic ones. The recent Dixit et al. model, trained by a VF dataset and supplemented by clinical data such as cup-to-disc ratio, intraocular pressure and central corneal thickness, succeeded in capturing VF trends and glaucoma progression and hence can be used as a diagnostic tool rather than a screening model that can actually supplement and confirm glaucoma diagnosis in specialized clinics with high specificity and accuracy (40). Furthermore, the DL model described by Fan et al. which was trained on a dataset of photographs from the ocular hypertension treatment study demonstrated diagnostic accuracy in the detection of primary open-angle glaucoma with a specificity that allows standardization of this model for the diagnosis of glaucoma in clinical trials (41). Also, more recent studies have focused on the diagnosis of glaucoma in myopic population (42) and to grade the severity of glaucoma in those with already confirmed diagnoses (43). Another important aspect in glaucoma diagnosis is documentation of disease progression over serial testing. This has been demonstrated by some models, including Belghith et al. (44) and Christopher et al. (45) model which demonstrated a high diagnostic specificity glaucoma progression detection on serial OCT images. Progression of glaucomatous changes in serial visual field tests can also be efficiently detected by AI models, as in (46), in which the application of progression of patterns (PAP) on the Gaussian mixture-model with expectation maximization (GEM) model has shown a high diagnostic accuracy for progressing glaucomatous visual field. Also, Sample et al.’s publication (47) has shown that AI models can detect progression and accurately differentiate true progressing changes from test-to-test variability.

## Discussion

According to the Ophthalmic Imaging and Intelligent Medicine Branch and the Intelligent Medicine Professional Committee of the China Medical Education Association guidelines on evaluation of ophthalmic AI research, AI-based ophthalmic diagnostic models are primarily evaluated based on sensitivity, specificity and accuracy indicators, other assessment parameters can include Kappa consistency coefficient, precision and receiver operating characteristic curve (AUC-ROC), among others (48). Clinical standards in ophthalmology often set benchmarks for these metrics, and AI models are evaluated against these standards to ensure their reliability. The reported performance across various modalities, such as OCT or fundus photography, contributes to the model’s versatility and practicality in real-world clinical settings. In our review, the AI models were categorized into screening models and diagnostic ones according to these indicators, particularly the sensitivity parameter in screening models, defined as the percentage of true positive subjects to all positive cases, and the specificity in diagnostic models, defined as the percentage of true negative samples to all negative samples. Additionally, all models reviewed in this paper were proven to have excellent performance on assessment and evaluation measures for ophthalmic AI research to ensure high quality, reliability and transparency in their design and development.

An AI model can be optimized for either screening or diagnostic tasks by adjusting its operating point. This involves finding the right balance between sensitivity and specificity, allowing customization based on the specific requirements of screening or diagnosis. The sensitivity-specificity tradeoff refers to the model’s ability to correctly identify positives (sensitivity) and negatives (specificity), and tuning helps tailor its performance to the desired emphasis in either screening or diagnostic applications (49). When tuning an AI model for screening, one should prioritize high sensitivity to minimize false negatives, ensuring a better chance of catching potential cases. However, the tradeoff may be an increase in false positives. This can be crucial when the goal is early detection or broad identification of potential issues. On the other hand, for diagnostic tasks, the emphasis may be on high specificity to minimize false positives, providing more confidence in the accuracy of the identified cases. The flexibility to adjust the operating point allows healthcare professionals or system administrators to align the AI model with the specific goals of the task at hand (50).

In view of the above, a sufficiently robust AI model, exhibiting high sensitivity and specificity, can be versatile enough to be applicable in both screening and diagnostic tasks. The dual strength of high sensitivity and high specificity ensuring effective identification of positives and minimizing false positives will make the model reliable for early detection in screening scenarios and accurate diagnosis in more detailed examinations. The concept implies that a well-balanced AI model can offer a practical and efficient solution for both broad identification and precise confirmation of glaucoma.

When discussing the economic aspects of glaucoma screening, we should delve into the consequences of sensitivity and specificity tradeoffs. A model with high sensitivity that detects actual positive cases early would potentially reduce the future burden of treating advanced stages of glaucoma. Early intervention can lead to more cost-effective and less invasive treatments compared to managing advanced cases (51). However, striving for high sensitivity may result in an increased number of false positives, where the model identifies cases that, upon further review, turn out not to be glaucoma. This triggers additional human involvement, clinic visits, and diagnostic tests, incurring costs in terms of both time and resources that could be allocated more efficiently. Therefore, finding the right balance is essential. A well-tuned AI model should minimize the number of missed positive cases while keeping false positives at an acceptable level. This approach optimizes the economic efficiency of glaucoma screening ensuring that the benefits of early detection outweigh the costs associated with false positives. It’s a delicate equilibrium that considers both the potential burden of treating late-stage glaucoma and the economic cost of false positives in terms of human time and effort.

While AI models can enhance efficiency and assist in the screening process, they are often viewed as tools to augment human capabilities rather than replace them (52). Human clinicians act as the final arbiter, incorporating AI-generated insights into their decision-making process. Our expertise remains crucial for several reasons. Firstly, we still need a clinical Judgment and consideration of various factors beyond what an AI model might analyze, such as patient history, comorbidities, and lifestyle factors. Also, some glaucoma cases can be complex, requiring a deep understanding of the clinical context. Human clinicians are adept at handling intricacies that may be challenging for AI models to interpret accurately. Moreover, effective communication with patients is vital in healthcare. Clinicians can explain diagnoses, treatment plans, and potential outcomes in a way that patients can understand, addressing their concerns and ensuring informed decision-making. Finally, ethical considerations involved in diagnosis and disease monitoring require human clinicians to navigate the complexity of patient preferences, cultural factors, and ethical guidelines (53). This collaborative approach ensures a balance between the efficiency of AI technology and the expertise, empathy, and holistic understanding brought by human clinicians in the field of glaucoma screening and diagnosis.

## Obstacles and challenges

Although the implementation of AI in the field of glaucoma diagnosis presents numerous advantages in screening and diagnosis of glaucoma, there are certain limitations to consider. AI algorithms have predominantly been trained and validated on specific patient groups or collections of datasets. This raises questions about the potential to generalize their outcomes to real-world populations where many confounding ocular and medical complexities exist rather than ideal-world situation. Furthermore, challenges persist in scenarios involving patients with anomalous, tilted or crowded optic discs, where misclassification could be a major concern (3). Another significant hurdle to the adoption of AI strategies in glaucoma ophthalmic field is what is called the “black box” phenomenon (54). This refers to the process of mental acceptance of outputs from a brand-new machine, the decision processing of which is vague or incomprehensible and whether to trust and largely apply such techniques instead of the traditional “old-school” methods. In general, robust large-scale population-based validation of these algorithms is imperative to confidently extend their use in general glaucoma diagnosis (2).

## Conclusion

The use of AI in the diagnosis of glaucoma holds immense promise for enhancing early detection, accuracy, and accessibility. While many models were proven to be successful as screening tools with high sensitivity to detect glaucoma, the development of diagnostic models with the utilization of multi-modal anatomical and functional datasets that can serve as glaucoma diagnostic models in specialized glaucoma care clinics remains a challenge. As research and development in this field continue, a synergy between AI and clinical expertise will pave the way for a more comprehensive approach to revolutionize glaucoma diagnosis and improve patient outcomes.

## Author contributions

MA: Methodology, Supervision, Validation, Writing – original draft, Writing – review & editing. SA: Conceptualization, Data curation, Formal analysis, Writing – review & editing. MB: Conceptualization, Project administration, Resources, Supervision, Writing – review & editing. AA: Conceptualization, Methodology, Supervision, Validation, Writing – review & editing. MA-A: Formal analysis, Software, Writing – review & editing.
